# Melatonin as a multifunctional modulator: emerging insights into its role in health, reproductive efficiency, and productive performance in livestock

**DOI:** 10.3389/fphys.2024.1501334

**Published:** 2024-12-05

**Authors:** Ali Afzal

**Affiliations:** ^1^ Animal Sciences Division, Nuclear Institute for Agriculture and Biology College (NIAB-C), Pakistan Institute of Engineering and Applied Sciences (PIEAS), Faisalabad, Pakistan; ^2^ School of Zoology, Minhaj University Lahore, Lahore, Pakistan

**Keywords:** melatonin, health, antioxidant status, animal performance, production potential

## Abstract

Melatonin, a pleiotropic hormone plays a vital role in enhancing livestock performance not only by regulating circadian rhythms but also by exhibiting antioxidant, immunomodulatory, and metabolic regulatory effects that collectively improve resilience, fertility, and productivity. Melatonin’s synthesis is predominantly influenced by light exposure, with increased production in darkness; however, factors such as diet and health status further modulate its levels. By helping animals adapt to environmental stressors, melatonin boosts immune responses, mitigates chronic illnesses, and optimizes production efficiency. Its regulatory influence extends to the hypothalamic-pituitary-gonadal (HPG) axis, enhancing hormone secretion, synchronizing estrous cycles, and improving embryo viability. This results in improved reproductive outcomes through the protection of gametes, increased sperm motility, and enhanced oocyte quality, all of which benefit the fertilization process. Additionally, melatonin positively impacts productive performance, promoting muscle growth, development, and optimizing milk yield and composition through its interaction with metabolic and endocrine systems. As ongoing research continues to uncover its broader physiological effects, melatonin supplementation emerges as a promising approach to improving livestock welfare, productivity, and sustainability in modern animal husbandry.

## 1 Introduction

Melatonin, a versatile hormone mainly synthesized by the pineal gland, has a unique lipophilic structure that enables it to cross biological membranes, including the blood-brain barrier. This characteristic facilitates its widespread distribution throughout the body, allowing melatonin to interact with both endocrine and non-endocrine tissues (Kopustinskiene and Bernatoniene, 2021). However, melatonin synthesis is not confined to the pineal gland; tissues like the retina, gastrointestinal tract (GIT), and immune cells also produce extra-pineal melatonin, reinforcing its systemic influence (Markus et al., 2021). In livestock, melatonin has gained prominence due to its extensive role in improving health, reproductive efficiency, and productive performance (Al-Hamedawi and Hatif, 2020; Yang et al., 2021; Abulaiti et al., 2023; Leyva-Corona et al., 2023). While traditionally recognized for regulating circadian rhythms, melatonin exerts broader physiological effects by enhancing immune function, reducing oxidative stress, and promoting overall wellbeing in livestock species (Deng et al., 2020; Yin et al., 2020).

The antioxidant action of melatonin is key to its positive health impacts on livestock (Jaworek et al., 2021). It neutralizes free radicals, reduces lipid peroxidation (LPO), and activates antioxidant enzymes like superoxide dismutase (SOD), catalase (CAT) and glutathione peroxidase (GPx), mitigating oxidative stress commonly encountered in high-production environments. These effects help prevent cellular damage, and lower the incidence of diseases such as mastitis and salmonellosis, thereby contributing to increase longevity and productivity in livestock (Li et al., 2020; Yao et al., 2020; Chen et al., 2022; Al-Jebory et al., 2024).

Melatonin’s impact on the immune system extends beyond its antioxidant properties. Through receptor mediated (RM) and non-receptor mediated (NRM) pathways, it influences inflammatory responses by regulating the release of cytokines, including interleukins (IL)-2, IL-6, and tumor necrosis factor (TNF)-α (Ferreira et al., 2021; Muñoz-Jurado et al., 2022). This regulation is particularly valuable in managing immune-related conditions in livestock animals.

In reproductive physiology, melatonin is essential for managing the HGP axis, improving the production of key reproductive hormones like gonadotropin-releasing hormone (GnRH), luteinizing hormone (LH), and progesterone (P_4_) (Wassem et al., 2022). In seasonal breeders melatonin influences estrous cycles, ovulation, and luteal activity, improving fertility outcomes (de Carvalho et al., 2021; Abulaiti et al., 2023; Yesilkaya and Erdem, 2024). Further, melatonin’s protective effects on ovarian tissues, through its antioxidant action, reduce oxidative stress and apoptosis, supporting higher pregnancy rates (Sun et al., 2020; Hashem et al., 2023). In male animals, melatonin modulates testicular function, improving semen quality and bolstering artificial insemination (AI) programs (Samir et al., 2020; Akar et al., 2024). Its impact on energy metabolism and mitochondrial function further promotes reproductive and productive efficiency, particularly under stress-inducing environments.

The role of melatonin also extends to enhance the productive performance of livestock by fostering growth, muscle development, and feed efficiency (Viola et al., 2023). By modulating fat deposition and muscle growth through key myogenic regulatory factors, melatonin improves feed conversion ratios. This results in greater weight gain and improved milk yield. Furthermore, its interaction with the intestinal microflora enhances nutrient absorption and immune responses, augmenting growth and overall performance in poultry and ruminants (Al-Samrai et al., 2023; Kanyar and Karadaş, 2023).

This study aims to deliver a thorough review of recent research and new insights into the impact of melatonin on livestock. By reviewing the latest scientific research, it aims to present current knowledge on how melatonin impacts livestock health, reproductive outcomes, and productive performance. Potential strategies for optimizing melatonin supplementation in livestock will also be discussed, alongside highlighting key opportunities for further investigation, aiding to the ongoing discussion on sustainable animal husbandry practices amid modern agricultural challenges.

## 2 Factors influencing melatonin synthesis

The synthesis of melatonin in livestock is influenced by a range of environmental, physiological, and management factors, each affecting production levels in unique ways. This complex regulation of melatonin synthesis is essential for aligning various physiological processes, such as reproductive cycles, immune responses, and stress resilience, with the environment. The factors detailed in Table 1 play significant roles in either enhancing or inhibiting melatonin production, depending on circumstances such as light exposure, seasonal changes, and stress (Hyder et al., 2017; Misztal et al., 2018; Zhao et al., 2019; Li H. et al., 2021).

**TABLE 1:** Factors affecting melatonin synthesis in livestock animals.

Factor	Effect on melatonin synthesis	Remarks
Photoperiod (Light Exposure)	Increased melatonin synthesis during darkness; reduced during light exposure	Melatonin is produced in the absence of light; artificial lighting can suppress production
Seasonal Variation	Longer nights (winter) increase melatonin synthesis; shorter nights (summer) decrease it	Strongly affects reproductive cycles and other physiological processes
Age of the Animal	Melatonin production decreases with age	Younger animals produce more melatonin than older animals
Species Differences	Varies by species; ruminants exhibit stronger seasonal effects	Some species (e.g., sheep, Goats) are more affected by photoperiod changes
Nutritional Status	Adequate tryptophan and antioxidants enhance melatonin synthesis	Diets rich in tryptophan and antioxidants support melatonin production
Stress Levels	High stress reduces melatonin synthesis	Stress activates the HPA axis, increasing cortisol, which inhibits melatonin
Hormonal Influences	Increased cortisol reduces melatonin synthesis	Hormones like prolactin can also modulate melatonin in reproductive cycles
Temperature and Climate	Cooler temperatures increase melatonin production; heat stress decreases it	Environmental temperature fluctuations can influence melatonin levels
Circadian Rhythm Disruption	Disruption of natural light cycles decreases melatonin synthesis	Common during transport or changes in housing systems, leading to misalignment with the internal clock

## 3 Sources of melatonin

Melatonin occurs naturally in various edible plants and plant-based products, making it an advantageous component in livestock diets. These plants not only contain melatonin but also its precursors, with varying concentrations significantly depending on the specific plant tissue (Table 2) (Tan et al., 2012). These edible sources of melatonin are prevalent across common forage crops and grains used in livestock production. Forages such as alfalfa, clover, and ryegrass, frequently consumed by ruminants, naturally supply melatonin that aids in reducing stress and fostering relaxation in animals, leading to improved health and productivity.

**TABLE 2:** Melatonin content in livestock feed sources.

Plant/Food	Amount of melatonin	Part/Organ of plant	Reference
Alfalfa	20–80 pg/g	Leaves and stems	Reiter and Tan (2002)
Ryegrass	5–15 pg/g	Leaves	Wu et al. (2021)
Clover	10–20 pg/g	Leaves	Bajwa et al. (2014)
Corn	8–12 pg/g	Kernels	Badria et al. (2002)
Oats	25–45 pg/g	Grain	Hattori et al. (1995)
Barley	15–30 pg/g	Grain	Badria et al. (2002)
Rice	80–150 pg/g	Bran	Hattori et al. (1995)
Soybeans	10–50 pg/g	Seeds	Reiter and Tan (2002)
Lentils	15–25 pg/g	Seeds	Aguilera et al. (2015)
Chickpeas	20–40 pg/g	Seeds	Reiter and Tan (2002)
Sunflower	10–30 pg/g	Seeds	Reiter and Tan (2002)
Flaxseeds	30–60 pg/g	Seeds	Reiter and Tan (2002)
Corn Silage	5–15 pg/g	Whole plant	Li et al. (2021c)
Grass Silage	10–20 pg/g	Whole plant	Li et al. (2021c)
Walnuts	3,000–4,000 ng/g	Nuts	Reiter et al. (2005)

## 4 Melatonin mechanism of action (RM and NRM)

Melatonin exerts its multifaceted effects on livestock through a combination of RM and NRM mechanisms, influencing various physiological processes. Its primary mode of action is through endocrine, autocrine, and paracrine pathways, largely facilitated by its binding to plasma membrane receptors and interactions with intracellular proteins (Samec et al., 2021). The distribution of melatonin receptors across different tissues and organs in livestock varies significantly. In mammals, including livestock animals, melatonin primarily engages with G-protein coupled receptors (GPCRs), such as melatonin receptor (MT) 1, MT2, and MT3, which are crucial for regulating processes like circadian rhythms, cardiovascular function, and immune responses (Boiko et al., 2022; Cecon et al., 2023). MT1 and MT2 receptors, in particular, play significant roles in livestock, where they regulate circadian rhythms and cardiovascular activity through inhibition of adenylate cyclase and modulation of phospholipase C signaling (Li M. et al., 2021; Samanta, 2022). MT3 receptors, which belong to the quinone reductase family, contribute to detoxification processes and oxidative stress reduction (Shabajee-Alibay et al., 2022).

Besides its effects through receptor interactions, melatonin also demonstrates important NRM effects, particularly in its role as a potent antioxidant. Melatonin’s ability to directly scavenge free radicals and activate antioxidant enzyme pathways underscores its protective capacity within cells. These actions help livestock combat oxidative stress, a common challenge in high-stress production environments (Purushothaman et al., 2020; Ikram et al., 2021). Melatonin also binds to transition metals, preventing the formation of harmful hydroxyl radicals, further supporting its antioxidant function (Galano et al., 2021). In livestock animals, melatonin is highly concentrated in mitochondria, where it protects vital cellular components—proteins, lipids, and DNA—from oxidative damage induced by free radicals during cellular respiration (Esteban-Zubero et al., 2023; Kennaway, 2023).

## 5 Health effects of melatonin in livestock

### 5.1 Source of circulating amino acids

Melatonin, synthesized from tryptophan, has garnered increasing interest due to its potential impact on circulating amino acids. Melatonin alleviated the impact of nutrient restriction on the levels of total amino acids and branched-chain amino acids during gestation in both small and large ruminants (Trotta et al., 2021; Swanson et al., 2022). Additionally, in these animals, melatonin exhibited a rescuing effect on nutrient restriction in various transport systems, including System A, System N, and anion amino acids (Swanson et al., 2022). This dual action of melatonin underscores its potential to modulate amino acid availability and transport systems, highlighting its significance in maintaining metabolic balance during gestation.

Research on melatonin as a therapeutic has expanded beyond gestational issues. In mammary glands cancer, melatonin diminished the impact of cancer on the levels of circulating amino acids. Specifically, aspartate, leucine, lysine, proline, serine, and valine concentrations were influenced by melatonin (Junior et al., 2022). This exhibits that melatonin effectively regulates amino acids in cancer, potentially inhibiting tumor growth by reducing the fuel source for cancer cells.

### 5.2 Endocrine modulations

The secretion patterns of melatonin are intricately linked to the metabolism of steroids and prostaglandins (PG) in livestock. In particular, luteal cells exposed to melatonin demonstrate a stimulatory effect on P_4_ hormone production (Bouroutzika et al., 2020; Pool et al., 2020). Melatonin supplementation has been shown to reduce PGF2 and estrogen (E_2_) levels in endometrial and hypothalamic cells, accompanied by a simultaneous decrease in the uterine contractile response to oxytocin (Wang et al., 2021a; Duan et al., 2022; Kacar et al., 2023). The interaction between melatonin and E_2_ receptors mirrors that of a selective E_2_ receptor modulator, potentially inhibiting E_2_ synthesis in steroidogenic tissues (Cos et al., 2014). Furthermore, melatonin has been linked to reduced activity and expression of aromatase, the enzyme involved in E_2_ production, as well as sulfatase, which affects E_2_ availability. This reduction might result in higher activity of E_2_ sulfotransferase, which produces E_2_ sulfate—a variant with diminished biological activity but a longer duration in the body (Jin et al., 2021).

Dietary supplementation of melatonin during the later stages of gestation has been linked to a reduction in both estradiol-17β and P_4_ concentrations (McCarty et al., 2018). This effect is attributed to the possible enhancement of cytochrome P450 1A enzymatic activity (Hwang et al., 2020; Singh M. et al., 2020; Hwang et al., 2021). A deficiency in estradiol production has been connected to traits resembling pre-eclampsia, suggesting that changes in estradiol metabolism due to melatonin exposure may influence utero-placental development during pregnancy (Sljivancanin Jakovljevic et al., 2020).

Exposure of bovine endometrial epithelial cells to estradiol results in decreased expression of melatonin receptor 1, whereas P_4_ treatment leads to an increase in this receptor’s expression (Brockus et al., 2016a). These findings underscore the complex relationship between the synthesis and metabolism of utero-placental steroids and PG and their influence on nutrient transport and uterine blood flow (Harman et al., 2023). Additionally, E_2_ is known to suppress adrenergic tone in the uterine arteries. Increased melatonin levels might lower E_2_ levels or sensitivity, potentially influencing the regulation of uterine blood flow, especially in problematic pregnancies (Edwards et al., 2020; Gaur and Purohit, 2020; Piotrowska-Tomala et al., 2022).

### 5.3 Impact on microbiome

**FIGURE 1:**
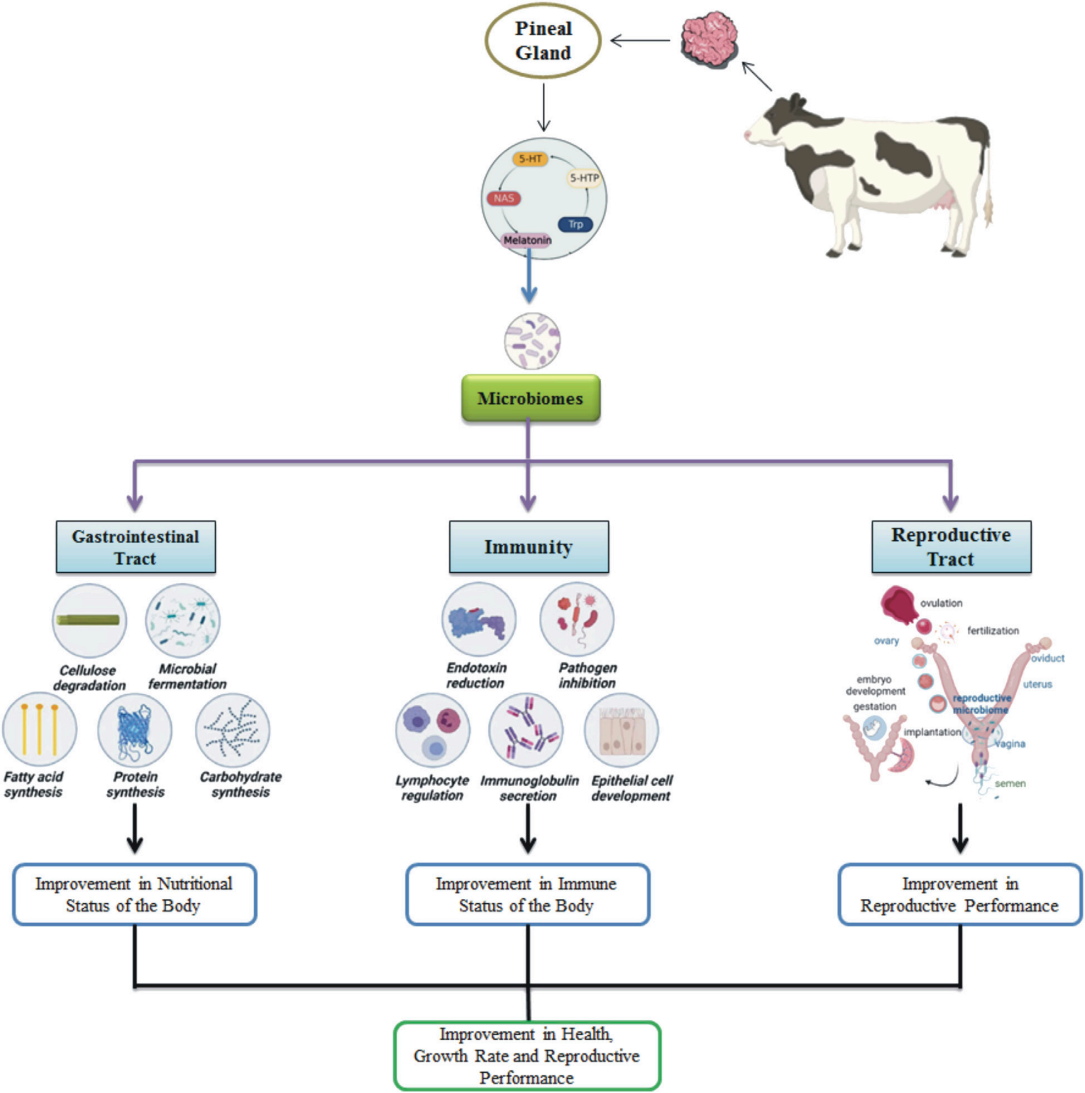
Melatonin effects on the microbiomes in the animal’s body, influencing gastrointestinal, immune, and reproductive functions.

The intricate interplay between immune system modulation and microbial fluctuations across the body has recently gained considerable attention in various livestock species. Harnessing the correlation between microbial presence and immune status has become an exciting frontier in livestock research. Melatonin exerts a profound influence on microbial populations of different systems in the body of animals, highlighting the vast scope of its biological impact. (Figure 1).

#### 5.3.1 GIT microbiome

Melatonin has significant potential in alleviating microbial dysbiosis (Gao et al., 2021). Its effects are mainly mediated through toll-like receptor 4 (TLR4), which plays a crucial role in pathogen-associated molecular pattern (PAMP) signaling, especially in relation to lipopolysaccharides (LPS) found in Gram-negative bacteria (Kim et al., 2020). Melatonin’s presence within the GIT is remarkable, with concentrations exceeding those in the pineal gland by up to 400-fold, reflecting the abundance of melatonin receptors and the enzymes required for its production in the gut (Kennaway, 2023). Furthermore, gut microbes exhibit circadian rhythms that mirror those of the host, significantly influencing their metabolic functions (Zheng et al., 2024). Given melatonin’s critical role in regulating the biological clock, it becomes evident that the circadian rhythms of gut microbes and their functions are intricately tied to melatonin.

The identification of rhythmic patterns in the ruminant gut microbiome has prompted additional research, suggesting that melatonin’s effects might stem from its presence in saliva. Salivary melatonin, known for its role in regulating inflammation, promoting antioxidant responses, and accelerating the healing of oral wounds (Elsherbini and Ezzat, 2020), exhibits circadian rhythms similar to those found in ruminal fluid and muscularis (Ouyang et al., 2021). This implies that melatonin present in saliva might affect microbial communities across the GIT through circadian variations. Indeed, the rhythmic changes in rumen microbial populations correspond with fluctuations in melatonin levels, with elevated melatonin associated with a higher relative abundance of the families Preovotellaceae and Muribaculaceae, and a reduction in Succininivibrionaceae and Veillonellaceae (Fu et al., 2023). These findings exhibit melatonin’s capacity to impact Gram-negative bacteria through cytokine production and metabolic regulation (Xue et al., 2023). Fluctuations in melatonin levels within the GIT affect crucial metabolic pathways, thereby impacting the predominant phyla in the rumen, such as Firmicutes, Proteobacteria, and Bacteroidetes (Fu et al., 2023).

#### 5.3.2 Reproductive tract microbiome

Melatonin has recently emerged as a promising regulator of the reproductive tract microbiome in livestock. The reproductive microbiome plays a vital role in fertility and overall reproductive efficiency (Hussain et al., 2021). In cows, melatonin supplementation has been shown to modulate bacterial populations within the uterus and vagina, fostering a more favorable environment for reproduction. This effect is attributed to melatonin’s potent anti-inflammatory and antioxidant properties, which may create optimal conditions for embryo development and implantation (Messman et al., 2021).

Beyond its influence on microbial composition, melatonin also impacts the immune response within the reproductive tract, which is intricately linked to the microbiome (Wang et al., 2021b). By enhancing immune function, melatonin indirectly supports a balanced microbiome, further promoting reproductive success. This dual role underscores the complex interplay between melatonin, the microbiome, and immune health, all of which are crucial for successful reproduction in livestock. Interestingly, melatonin’s effects on the reproductive tract microbiome vary across livestock species (Cosso et al., 2021). Factors such as physiological state, age, and environmental conditions likely contribute to these variations. Understanding these complexities is essential for developing tailored melatonin supplementation strategies that maximize reproductive health benefits across diverse livestock populations.

### 5.4 Immunomodulation

Melatonin, recognized for its immune-boosting and anti-apoptotic effects, is crucial in regulating immune responses, especially by boosting the T helper 1 immune pathway (Esquifino et al., 2004). While its primary function lies in regulating circadian rhythms, melatonin also exerts significant secondary effects on the immune system, including upregulating cytokine production, promoting T cell propagation, stimulating natural killer (NK) cell activity, amplifying antigen presentation, and maintaining a balanced cluster of differentiation 4 (CD4) to CD8 immune cell ratio (Maestroni, 2001; Baltaci et al., 2018; Castellano and Molinier-Frenkel, 2020; Tune et al., 2020). Although studies on the use of melatonin supplements for livestock are still in the preliminary phase, it has shown promise in enhancing reproductive performance and mitigating stress or trauma-induced immunosuppression (Bouroutzika et al., 2021; Paulino et al., 2022).

Disruption of melatonin synthesis, whether through constant light exposure or the use of β-adrenergic blockers, results in a weakened immune response to antigens (Hanoun et al., 2015; Horodincu and Solcan, 2023). This immune suppression is characterized by an impaired primary antibody response, reduced immune cell populations in the thymus and spleen, and diminished lymphocyte proliferation. However, the administration of melatonin has been shown to reverse these immunosuppressive effects (Chang et al., 2020). Additionally, melatonin has been found to enhance vaccine efficacy, with animals receiving melatonin exhibiting a stronger antibody response post-vaccination, indicative of a more robust immune defense (Regodón et al., 2012; Cardinali et al., 2021; Wang S. et al., 2021). Further research into melatonin’s immune-stimulatory roles has revealed its complex interactions with cellular and cytokine profiles in both humoral and innate immune responses (Hosseini et al., 2021).

### 5.5 Impact on disease treatment

Melatonin treatment in *Trypanosoma cruzi* infections enhances the population of CD4^+^ CD28-negative T cells while simultaneously reducing CD28-negative cells within both the CD4^+^ and CD8^+^ subsets. This immunomodulatory effect is further reflected in the thymus, where melatonin decreases thyrotropin receptor antibody (TRAb) levels, aiding in the restoration of thymus size and thymocyte populations, essential for maintaining immune function (Brazao et al., 2020). Beyond its immune-boosting effects, melatonin’s potent anti-inflammatory properties offer significant protection against mastitis in dairy animals, helping to prevent the inflammatory damage commonly associated with this condition (Yao et al., 2020; Li and Sun, 2022).

In metabolic disorders like diabetes, melatonin demonstrates its antioxidant capabilities by normalizing malondialdehyde (MDA) and myeloperoxidase (MPO) levels while reducing cleaved caspase-3 expression, which signals its role in mitigating cellular damage (Abdulwahab et al., 2021). These protective effects extend to trauma-induced pulmonary issues, where melatonin improves total antioxidant capacity (TAC) and reduces organ damage, showcasing its broad therapeutic potential (Ates et al., 2022).

Melatonin’s influence is equally significant in the context of liver diseases, such as ischemia-reperfusion injury (IRI), non-alcoholic fatty liver disease (NAFLD), and cirrhosis, where it modulates the nitrogen oxide (NOx) and nuclear factor kappa B (NF-κB) pathways to alleviate oxidative damage and promote tissue recovery (Zhang C. et al., 2021; Esteban-Zubero et al., 2023). Similarly, in acute pancreatitis, melatonin effectively prevents LPO, while in kidney injury, it prevents cytotoxic impairment, highlighting its organ-protective role (Ahsen et al., 2014; Karabulut-Bulan et al., 2015). Moreover, its neuroprotective effects are evident in cases of brain damage, stroke, and ischemia, where melatonin mitigates inflammation, reduces edema, and promotes cell survival, further emphasizing its wide-ranging therapeutic applications across various systems (Mihardja et al., 2020; Wongchitrat et al., 2021).

### 5.6 Antioxidant properties

#### 5.6.1 Mechanisms of oxidative stress reduction

Melatonin is a potent antioxidant with the unique ability to dissolve in both water and fats, enabling it to function in various environments, such as within cell interiors, body fluids, membranes, and organelles (Zarezadeh et al., 2022). It outperforms conventional antioxidants like vitamins E and C in mitigating oxidative stress. Unlike typical antioxidants, which neutralize only one or a few reactive oxygen species (ROS), melatonin can neutralize up to 10 ROS molecules (Marchena et al., 2020). This remarkable capacity is attributed to its ability to stimulate key antioxidant enzymes, including SOD, CAT, GPx, and GSH, particularly when given in doses between 0.1 and 20 mg/kg per day (Table 3) (Baydas et al., 2002; Cruz et al., 2009; Nayki et al., 2016; Taghizadieh et al., 2016; Olayaki et al., 2018; Aranarochana et al., 2021; Bashandy et al., 2021; Colares et al., 2022; Abdel-Razek et al., 2023; Essawy et al., 2023).

**TABLE 3:** Impact of melatonin on various antioxidant defense mechanisms.

Dose	Administration Route	Antioxidant component	Response	References
10 mg/kg/day	Intraperitoneal	MDA	Significant Decrease (+)	Abdel-Razek et al. (2023)
		SOD	Significant Increase (+)	
		GPx	Slight Increase (++)	
20 mg/kg/day	Oral	MDA	Significant Decrease (++)	Colares et al. (2022)
		SOD	Significant Increase (++)	
10 mg/kg/day	Intraperitoneal	SOD	Significant Increase (++)	Essawy et al. (2023)
		GPx	Significant Increase (++)	
		CAT	Significant Increase (++)	
8 mg/kg/day	Intraperitoneal	GPx	Significant Increase (++)	Aranarochana et al. (2021)
		CAT	Significant Increase (++)	
		MDA	No Significant Difference	
2.5–5 mg/kg/day	Intraperitoneal	SOD	Significant Increase (++)	Bashandy et al. (2021)
		MDA	Significant Decrease (++)	
		CAT	Significant Increase (++)	
		GSH	Significant Increase (++)	
4–10 mg/kg/day	Oral	SOD	Significant Increase (++)	Olayaki et al. (2018)
		GPx	Significant Increase (++)	
		GSH	Significant Increase (++)	
		MDA	Significant Decrease (++)	
20 mg/kg/day	Intraperitoneal	SOD	Significant Increase (++)	Nayki et al. (2016)
		CAT	Slight Increase (+)	
		TOS	Significant Decrease (++)	
		TAS	Significant Increase (++)	
20 mg/kg/day	Intraperitoneal	GPx	Significant Increase (++)	Taghizadieh et al. (2016)
		SOD	Significant Increase (++)	
		MDA	Significant Decrease (++)	
0.5 mg/kg/day	Intraperitoneal	GSH	Significant Increase (++)	Cruz et al. (2009)
		SOD	Significant Increase (++)	
		MDA	Significant Decrease (++)	
		CAT	Significant Increase (++)	
		GPx	Significant Increase (++)	
0.1 mg/kg/day	Intraperitoneal	GPx	Significant Increase (++)	Baydas et al. (2002)

MDA: malondialdehyde; SOD: superoxide dismutase; GPx: Glutathione Peroxidase; GSH: reduced.

Glutathione CAT: catalase; TOS: total oxidative status; TAS: total antioxidant status.

Note: “Slight Increase (+)” indicates a minor effect, while “Significant Increase (++)” indicates a stronger impact.

Melatonin not only increases the mRNA levels of these antioxidant enzymes but also inhibits pro-oxidant enzymes such as nitric oxide synthase (Monteiro et al., 2024). By altering membrane fluidity, melatonin safeguards cell membranes from oxidative harm and eliminates free radicals before they damage lipids and proteins, all without promoting pro-oxidant effects (Kopustinskiene and Bernatoniene, 2021). It acts as an effective scavenger of free radicals and an electron donor, neutralizing various reactive species like hydroxyl radicals, hydrogen peroxide, and nitric oxide (Ahmadi and Ashrafizadeh, 2020). When melatonin interacts with hydroxyl radicals, it produces 3-hydroxymelatonin, which is later excreted through urine. Its metabolites, such as AMK and AFMK, display even greater antioxidant potency (Galano and Reiter, 2018; Zarezadeh et al., 2022).

#### 5.6.2 Initiation of antioxidant response components

Recent discoveries highlight melatonin’s pivotal role in activating the antioxidant response element (ARE) system, which triggers the transcription of numerous antioxidant proteins and enzymes to neutralize ROS and support essential protein transport (Vriend and Reiter, 2015). Central to this process is the Nuclear Factor Erythroid 2–Related Factor 2 (Nrf2)-ARE signaling pathway, which significantly enhances the activity of key antioxidant enzymes. This pathway provides a protective mechanism that shields livestock from various diseases, helping to preserve their productive potential (Zhang W. et al., 2018; Dong et al., 2020).

**FIGURE 2:**
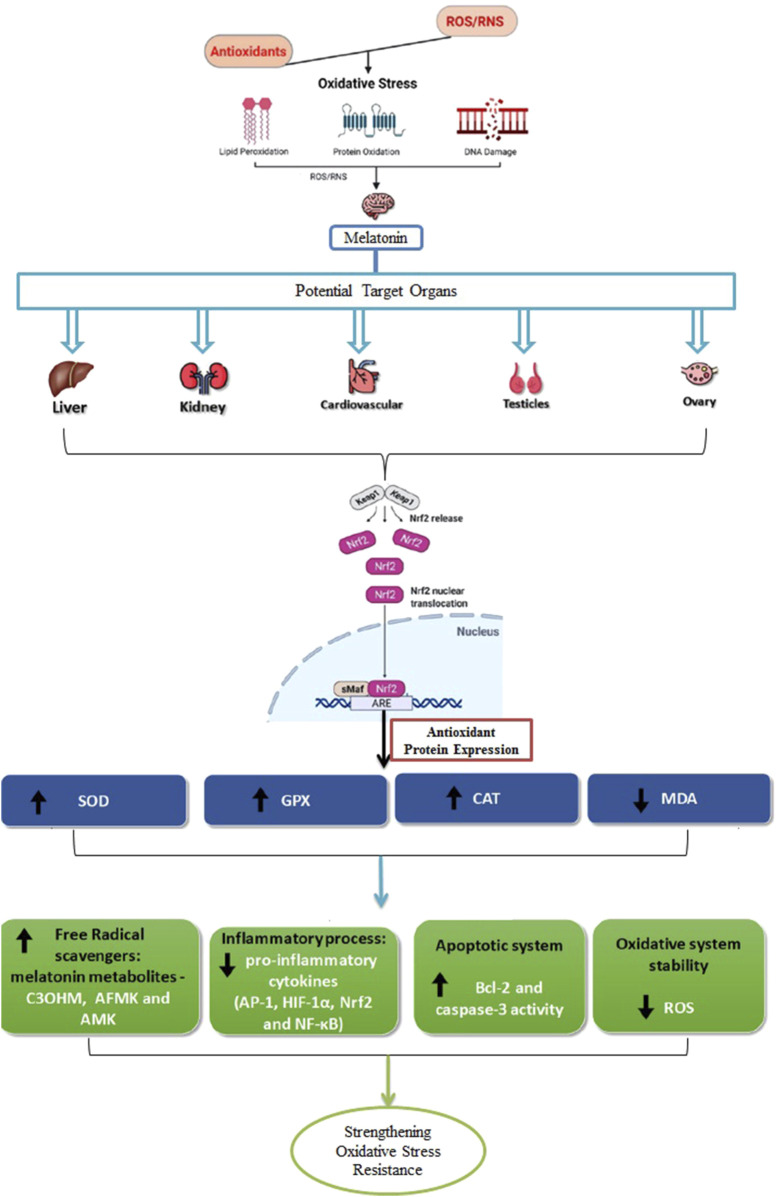
Antioxidant activity of melatonin across various tissues. It plays a role in receptor-independent pathways that result in the formation of metabolites like cyclic 3-hydroxymelatonin (C3OHM), N1-acetyl-N2-formyl-5-methoxykynuramine (AFMK), and N1-acetyl-5-methoxykynuramine (AMK). In addition to its antioxidant effects, melatonin modulates inflammatory responses by targeting key regulators, including activator protein 1 (AP-1), hypoxia-inducible factor 1α (HIF-1α), Nrf2, and NF-κB. It also impacts apoptotic pathways by interacting with proteins such as B-cell lymphoma 2 (Bcl-2) and contributes to maintaining redox homeostasis by reducing the generation of ROS. This leads to an upregulation of antioxidant enzymes like SOD, GPx, and CAT, while decreasing MDA levels, an indicator of LPO. Due to its amphiphilic nature, melatonin can easily cross tissue barriers, enabling it to exert protective effects on organs such as the liver, kidneys, cardiovascular system, as well as the reproductive organs like the testicles and ovaries.

Under oxidative stress, melatonin elevates cellular Nrf2 levels, which is crucial for its antioxidant function. Melatonin facilitates the nuclear translocation of the Nrf2 transcription factor and promotes its interaction with ARE, thereby amplifying the expression of antioxidant enzymes (Figure 2) (Deng et al., 2016). Further, melatonin has been demonstrated to reduce stress-related damage by safeguarding the hippocampus through the regulation of the Nrf2/Heme Oxygenase-1 (HO-1) pathway. Although research is still expanding, melatonin is also thought to increase the concentration of inhibitor of nuclear factor kappa B (IκB) alpha, an inhibitor of NF-κB, suggesting an additional mechanism by which it regulates inflammatory responses (Rajput et al., 2017).

Moreover, melatonin reduces the levels of nitrites, inducible nitric oxide synthase (iNOS), cyclooxygenase-2 (COX-2), and microsomal PG E synthase-1 (mPGES-1), while also preventing the translocation of NF-κB in peritoneal macrophages. By lowering pro-inflammatory mediators and enhancing HO-1 expression through NF-κB, p38 mitogen-activated protein kinase (MAPK), and Nrf2 signaling, melatonin shows significant potential as a therapeutic agent for conditions involving macrophage over-activation (Deng et al., 2020).

#### 5.6.3 Protection from harmful impacts of chemical agents inducing oxidative stress

Melatonin acts as a safeguard against the detrimental effects of various chemical compounds that induce oxidative stress. For instance, dizocilpine maleate (MK-801), a compound known to induce oxidative damage in the prefrontal cortex, leads to psychotic symptoms, but melatonin can counteract these effects. Similarly, cadmium negatively impacts male reproductive health; however, melatonin alleviates this toxicity by lowering MDA levels, boosting SOD activity, increasing GSH, and elevating pro-inflammatory cytokines like TNF-alpha and IL-1 beta (Venditti et al., 2021).

Melatonin also protects the cerebellum from damage caused by acrylamide by reducing LPO, boosting antioxidant enzyme activities, and lessening DNA damage (Ozturk et al., 2023). The cerebellum, which is highly susceptible to chemical insults, often undergoes neuron loss and organ shrinkage during development. Lead (Pb) is another toxicant that causes oxidative stress and neurotoxicity; however, melatonin (at 10 mg/kg) has been shown to reduce LPO and protect the cerebellum from Pb-induced toxicity (Bazrgar et al., 2015). Additionally, melatonin exhibits neuroprotective properties against ethanol toxicity in the cerebellum and lowers plasma homocysteine (Hcy) levels, further strengthening its role as a neuroprotective agent (Bagheri et al., 2024).

#### 5.6.4 Shielding against radiation-induced damage

Animals are exposed to various forms of radiation, against which melatonin provides protection. For instance, tropical animals frequently encounter ultraviolet (UV) radiation. When these animals were treated with melatonin after UV exposure, there was a marked increase in the activity of antioxidant enzymes such as SOD, CAT, and GPx. This enhanced enzyme activity neutralized free radicals, reducing the damage caused by UV radiation. Although UV radiation can indirectly harm spleen tissue, melatonin treatment aided in restoring balance and preventing splenocyte apoptosis, thus maintaining organ function (Goswami and Haldar, 2014). Melatonin also functions as an antioxidant in testicles exposed to microwaves, helping to alleviate oxidative stress and reduce DNA fragmentation (Özgen et al., 2023).

### 5.7 Effects on physiological stress markers

**FIGURE 3:**
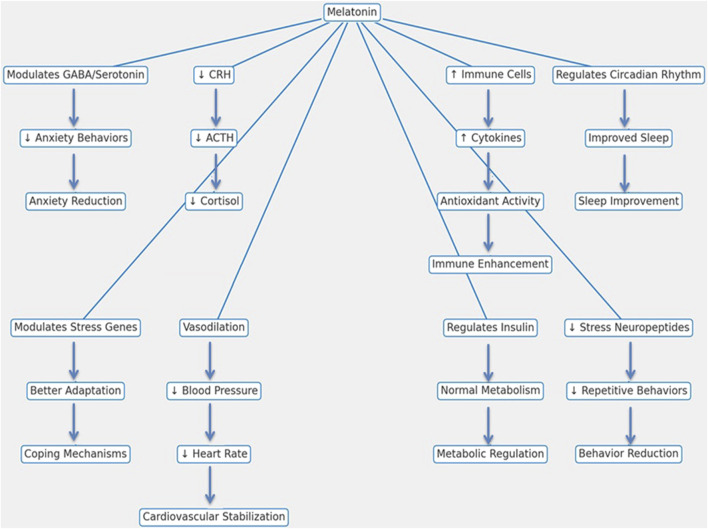
Mechanistic pathways of melatonin in reducing stress and enhancing physiological functions in livestock animals.

Livestock animals are routinely exposed to a range of environmental, physiological, and psychological stressors, which can negatively impact their health, productivity, and overall welfare (Johnson, 2018; Chauhan et al., 2021). Elements like handling practices, environmental changes, shifts in herd dynamics, and illness can impair immune function, resulting in chronic diseases, weight loss, and reduced production, all of which have considerable economic impacts. In this regard, melatonin, known for its immune-stimulatory properties, plays a vital role in reducing stress by regulating both physiological and psychological responses in livestock (Figure 3).

Melatonin primarily alleviates stress by modulating the hypothalamic-pituitary-adrenal (HPA) axis, an essential part of the body’s stress response system. It reduces the secretion of corticotropin-releasing hormone (CRH) and adrenocorticotropic hormone (ACTH), leading to lower cortisol production, which is a major marker of physiological stress (Kumar and Singh, 2021). Further, melatonin affects the autonomic nervous system by encouraging vasodilation, lowering heart rate and blood pressure, and contributing to stable cardiovascular function (Imenshahidi et al., 2020; Zuo and Jiang, 2020).

Melatonin interacts with neurotransmitter systems, including gamma-aminobutyric acid (GABA) and serotonin, to produce anxiolytic effects, reducing restlessness and aggression, especially during stressful events like transport and weaning (Xu et al., 2023). This reduction in anxiety enhances social behavior and improves interactions within the herd. Melatonin is also vital in managing the sleep-wake cycle and enhancing sleep quality, which is important for effective stress recovery. Better sleep enhances resilience against environmental stressors such as changes in housing and climate variations (Domple et al., 2017). Beyond its role in stress mitigation, melatonin contributes to metabolic regulation by interacting with insulin and other metabolic hormones, thereby stabilizing glucose and lipid metabolism and preventing stress-induced metabolic disorders (Samir et al., 2023). It also modulates gene expression in immune cells, boosting immune function, controlling inflammation, and supporting cell proliferation, all of which enhance the resilience of livestock animals (Wang et al., 2022). Levels of stress-related neuropeptides and hormones that are associated with abnormal behaviors such as pacing and excessive grooming are also reduced by melatonin in stressed animals (Zhang H. et al., 2021). By mitigating stress across multiple physiological systems, melatonin ultimately improves both welfare and productivity in livestock.

## 6 Reproductive performance

### 6.1 Mediation of reproduction

Melatonin regulates reproductive function by acting at both the hypothalamic and pituitary levels through highly expressed receptors (Wassem et al., 2022). Melatonin may affect reproduction by interacting with the hypothalamus through RM mechanisms and uptake. It reduces GnRH activity in the pituitary and lowers hypothalamic secretion by 45% by activating protein kinase (PK) A, PKC, and MAPK pathways. This suppression of gonadotropin release can help control the timing of puberty, as a drop in melatonin levels below a specific threshold prompts the hypothalamus to trigger reproductive changes (Rijal et al., 2020; Ding et al., 2021; Charif et al., 2022).

The pathway connecting the supra-chiasmatic nucleus (SCN) and the pituitary is crucial for reproduction in seasonally breeding mammals (Tolla and Stevenson, 2020). Information about light and dark cycles is relayed from the SCN to the pineal gland through a complex network of synapses, resulting in varying patterns of melatonin secretion that translate light cues into hormonal rhythms. Animals with SCN lesions or those that have had their pineal glands removed are unable to produce these photoperiodic responses, leading to disruptions in reproductive function (Carcangiu et al., 2014). Variations in day length serve as a “calendar” for many species, timing reproductive activity to coincide with periods of favorable energy availability and climatic conditions that enhance offspring survival. This conversion of environmental signals into the neuroendocrine system is facilitated by variations in nocturnal melatonin secretion (Giannetto et al., 2020).

Alterations in melatonin secretion resulting from changes in day length are consistent across different species but have varying impacts on reproductive function depending on the seasonal reproductive patterns. For short-day breeders like sheep and goats, elevated nocturnal melatonin levels stimulate the reproductive system, whereas in long-day breeders, these elevated levels suppress reproductive activity. Melatonin exerts its influence on reproduction both centrally, through its actions on the hypothalamus and pituitary, and directly in the gonads, which are not only targets of melatonin but also sites of its production (Singh P. et al., 2020; Tölü et al., 2022).

### 6.2 Influence of melatonin on ovarian activity

#### 6.2.1 Granulosa cells (GC)

Follicular atresia significantly affects bovine reproductive performance, as it heavily depends on the health of GCs for ovarian follicle development. Disturbances in these cells, whether through apoptosis, autophagy, cell cycle arrest, or accumulation of ROS, can lead to follicular atresia (Wang H. et al., 2018; Ma et al., 2019; Wang Y.-X. et al., 2021). Additionally, alterations in steroid hormone synthesis further influence GC function. Melatonin, known for its ROS-scavenging properties and cellular regulatory abilities, plays a critical role in mitigating follicular atresia by reducing ROS levels and inhibiting apoptosis in GCs through various pathways (Xie et al., 2022).

In the initial phases of follicular atresia, apoptosis predominantly targets the inner layer of GCs, while the cumulus-oocyte complex and outer GCs remain largely unaffected, highlighting the selective nature of this process (Rajin et al., 2022). GCs are crucial for supporting and maintaining follicle growth, and their physiological condition greatly influences the fate of the follicle (Yuan et al., 2019). Mitochondria, as a primary source of ROS, contribute to mitochondrial swelling and apoptosis when ROS levels become excessive. Melatonin helps maintain antioxidant enzyme activity and neutralizes reactive oxygen by regulating ER oxidoreductin 1 (ERO1) and enhancing the activities of antioxidant enzymes (Fernández et al., 2015; Tamura et al., 2020).

Melatonin has been found to mitigate oxidative stress and apoptosis in bovine ovarian GCs caused by β-zearalenol (Yang et al., 2019). The protective effects of melatonin are modulated by its receptors, MT1 and MT2, as inhibition of these receptors can diminish melatonin’s benefits and interfere with cell cycle regulation (Gobbi and Comai, 2019). Melatonin’s influence also varies with environmental conditions such as temperature and oxygen concentration. For example, at 37.5°C and 5% O_2_, low concentrations of melatonin promote cell proliferation, while at 40°C, higher concentrations have the same effect (Zeebaree et al., 2018). This temperature-dependent response highlights melatonin’s potential in mitigating heat stress. Nonetheless, differences in physiological conditions and natural melatonin production make the reliable and effective use of supplemental melatonin challenging, highlighting the need for comprehensive data to guide evidence-based practices.

#### 6.2.2 Follicles

Melatonin supports the development of bovine secondary follicles through membrane-bound receptors, whereas its antagonist, luzindole, inhibits these effects and decreases the expression of antioxidant enzymes in cultured follicles (Paulino et al., 2022). Additionally, melatonin stimulates follicular angiogenesis by increasing VEGF expression, which is crucial for follicular development (Tao et al., 2021). In theca cells, which exclusively express the MT2 receptor, melatonin inhibits androgen biosynthesis and slows ovarian atresia and aging by reducing apoptosis and regulating cell proliferation through the PI3K/Akt pathway (Wang S. et al., 2018; Ma et al., 2023). Abnormal melatonin levels in theca interna cells have also been linked to the development of follicular cysts in sows (Qin et al., 2022).

Beyond these roles, melatonin’s antioxidant properties support oocyte quality and enhance subsequent embryonic development, underscoring its potential in assisted reproduction. Studies have identified MT1 and MT2 mRNA in porcine cumulus-oocyte complexes, revealing that melatonin modulates GC function through MT2, which enhances cumulus expansion and embryonic development (He et al., 2016; Lee et al., 2018). Melatonin is critical for preventing age-related defects in germline-soma communication and aids in the transfer of antioxidant molecules from cumulus cells, preserving oocyte quality (Zhang H. et al., 2022).

Melatonin also shows promise as a pharmacological agent against endocrine disruptors such as Bisphenol A (BPA) and Bisphenol S (BPS), which impair follicular growth and steroidogenesis. It mitigates these harmful effects by increasing estradiol production, promoting GC proliferation, and protecting against mitochondrial apoptosis during oocyte maturation (Park et al., 2018; Wu et al., 2018; Berni et al., 2019). Its protective effects extend to other toxic substances, such as Aflatoxin B1 (AFB1), which induces follicular atresia and oxidative stress. Melatonin’s antioxidant properties and ability to inhibit apoptosis provide effective protection against AFB1-induced toxicity (Cheng et al., 2019).

Furthermore, melatonin enhances results in ovarian tissue cryopreservation and is vital for reproductive processes and blastocyst implantation in various mammalian species. It achieves this by regulating apoptotic mechanisms, enhancing adhesion protein expression, and protecting against oxidative stress that compromises embryo quality and pregnancy success (Najafi et al., 2023). The detection of melatonin in follicular fluid, along with reduced levels in cases of polycystic ovary syndrome (PCOS), further emphasizes its essential role in ovarian function and oocyte maturation (Shi et al., 2009).

#### 6.2.3 Oocyte cells

The quality of oocytes is essential for reproductive success in female animals and is a key factor in ruminant embryo transfer. Fresh embryos generally lead to significantly higher live birth rates compared to those that have been cryopreserved (Insogna et al., 2021). Cryopreservation of oocytes has long been challenging due to issues with survival, fertilization, and developmental rates (Chen et al., 2003). To overcome these challenges, enhancing the quality of oocytes cultured *in vitro* before cryopreservation is crucial, and melatonin has proven to be a promising approach.

**FIGURE 4:**
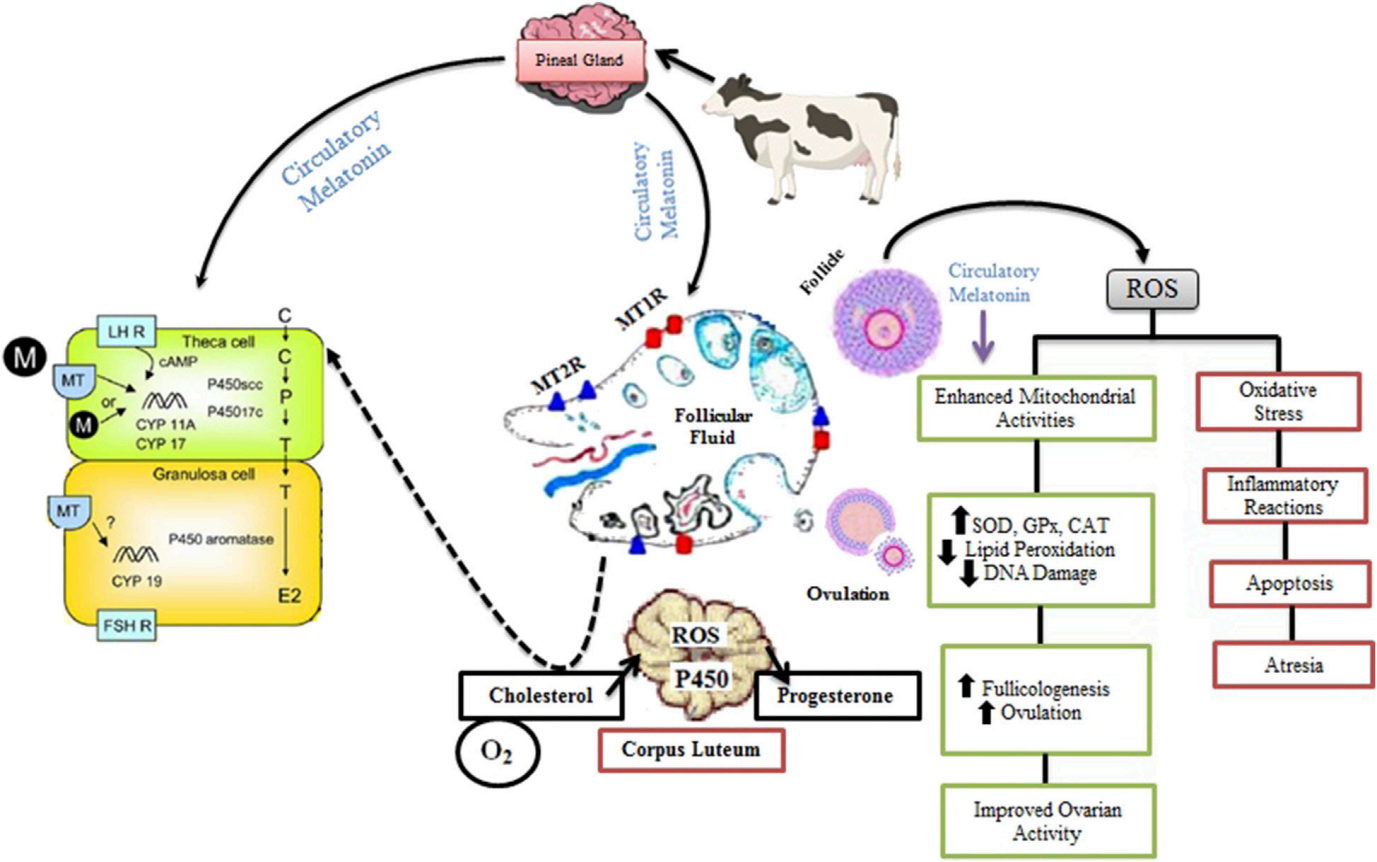
Role of Melatonin in Ovarian Function and Folliculogenesis in Livestock. Melatonin from circulation is taken up by the theca and granulosa cells of the follicle, where it interacts with its receptors, MT1R and MT2R, within the ovarian follicular fluid. This interaction stimulates the expression of LH and FSH mRNA, promoting hormone maturation and enhancing steroidogenic enzyme activities, such as those catalyzed by P450 aromatase, P450scc, and CYP17, which are responsible for synthesizing P_4_ and E_2_. During the process of follicular rupture, ROS are generated as a result of inflammatory reactions, leading to ovulation and further steroidogenesis, particularly the synthesis of P_4_ in the CL through monooxygenase reactions. Melatonin acts as a free radical scavenger, mitigating ROS-induced oxidative stress by boosting antioxidant enzymes like SOD, GPx, and CAT. This reduces LPO and DNA damage, while balancing ROS levels and antioxidative activity. By regulating mitochondrial function and minimizing oxidative damage, melatonin supports healthy follicle development, ovulation, and improved ovarian function, preventing detrimental effects such as apoptosis and follicular atresia. Ultimately, melatonin promotes the maturation of a healthy ovum, enhancing reproductive efficiency.

Studies show that melatonin can notably improve the developmental potential of oocytes, both *in vitro* and *in vivo* (Sananmuang et al., 2020). In cattle, melatonin improves oocyte developmental competence and embryonic growth by reducing ROS levels (Figure 4) (Gutiérrez-Añez et al., 2021). It also mitigates ROS in heat-stressed oocytes, increases maturation rates, boosts the proportion of embryos developing into blastocysts, and upregulates genes related to mitochondrial function (Yaacobi-Artzi et al., 2020). Melatonin also safeguards bovine oocytes from damage inflicted by harmful agents like paraquat, thus preserving their developmental potential (Pang et al., 2019).

Substantial evidence underscores melatonin’s role in advancing oocyte development in bovines. It boosts the production of antioxidant enzymes via specific membrane and nuclear receptors, aiding in the removal of ROS. In cumulus-oocyte complexes, acetylserotonin O-methyltransferase (ASMT) may play a role in melatonin synthesis (El-Raey et al., 2011). By reducing oxidative stress via the MT1 membrane receptor, melatonin preserves spindle body function, a critical factor for oocyte development.

In cattle, administering melatonin from days 190–262 of gestation enhanced uterine blood flow, probably due to its impact on steroid metabolism (Brockus et al., 2016b). Melatonin also positively influenced estradiol metabolism, improving utero-placental development (Lemley and Vonnahme, 2017). During estrus synchronization and AI in cattle, external melatonin significantly elevated P_4_ levels, boosted antioxidant enzyme activity, and reduced MDA concentrations in the blood, resulting in a marked improvement in pregnancy rates (Guo et al., 2021).

#### 6.2.4 Corpus luteum (CL)

A functional melatonergic system is essential for luteal function in mammals (Wang et al., 2021a). ROS, primarily generated from normal metabolic processes, are involved in both luteogenesis and luteolysis (Mierzejewski et al., 2023). During cholesterol transport for P_4_ synthesis and in the regression phase, LPO can damage the luteal plasma membrane, leading to impaired luteal function (Taketani et al., 2011; Cruz et al., 2014; Xu et al., 2021; Al-Shahat et al., 2022). Melatonin safeguards luteinizing GCs in the ovulatory follicle from ROS, boosts P_4_ production after ovulation, and helps prevent premature luteolysis in the newly developed CL. Elevated indolamine levels during the luteal phase underscore melatonin’s direct involvement in these processes. High concentrations of melatonin synthesis enzymes and receptor expression suggest that this site may serve as a key area for hormone synthesis and regulation (Xiao et al., 2018).

Melatonin deficiency in small ruminants disrupts follicular and luteal dynamics, resulting in decreased P_4_ synthesis (Kárpáti et al., 2023). In these animals, melatonin enhances P_4_ secretion by regulating autophagy through the AMPK/mTOR pathway (Duan et al., 2024). In equine corpus lutea, both MT1 receptor mRNA and protein are present. Melatonin reduces P_4_ production and P450scc expression in a dose-dependent manner. This inhibition can be reversed by luzindole, a non-selective melatonin receptor antagonist, indicating that functional melatonin receptors are present in luteal cells (Pedreros et al., 2011).

Melatonin’s role in luteal function has been evaluated during early pregnancy, given the endocrine organ’s importance in the initial stages of gestation (Verteramo et al., 2022). In heat-stressed cows, melatonin improves luteal hemodynamics (Abdelnaby and Abo El-Maaty, 2021). Local melatonin synthesis in the luteal cells of pregnant animals suggests a paracrine or autocrine role (Zhang et al., 2022b). In these animals, luteal cells during pregnancy show hormone receptor expression and increased P_4_ levels that correlate with melatonin concentration, mediated by upregulated P450scc and steroidogenic acute regulatory (StAR) protein (Zhang Y. et al., 2018). Moreover, melatonin stimulates GnRH and LH production in the luteal cells of pregnant animals, indicating a regulatory role through these hormones (Zhang et al., 2022c). Administering melatonin boosts the expression of genes related to pregnenolone synthesis, supports the development of the CL, aids in embryonic implantation, and enhances uterine receptivity during early pregnancy.

## 7 Melatonin influence on testicular function, spermatogenesis, and semen cryopreservation in livestock

Cryopreservation is vital for the long-term preservation of gametes in ruminants such as cattle and sheep, which is essential for genetic improvement and the conservation of endangered species. Although ROS are necessary for sperm capacitation, excessive ROS levels can cause damage to sperm morphology and DNA integrity through oxidative stress (Medrano et al., 2017; Ofosu et al., 2021). Melatonin by alleviating oxidative stress during sperm freezing, improves the quality of sperm after thawing (Appiah et al., 2019; Shahat et al., 2022).

Incorporating 1 mM melatonin into semen extenders improves sperm quality and mitigates the effects of heat stress. Additionally, melatonin implants significantly reduce the prevalence of morphologically abnormal sperm, increase motility, and boost total protein and cholesterol levels in seminal plasma (Inyawilert et al., 2021; Shahat et al., 2023). Optimal melatonin concentrations vary depending on the application: 10⁻³ M is recommended for semen cryopreservation, while 10⁻⁷ M is effective for enhancing oocyte maturation rates and increasing blastocyst numbers in in vitro fertilization (IVF). Medium concentrations of melatonin (0.25 mg/mL) yield the best results in terms of sperm motility and antioxidant indicators (Ramadan et al., 2019; Su et al., 2021).

Aside from influencing semen quality, melatonin is essential for spermatogenesis, as it regulates testicular function via the HPG axis. It delays puberty by decreasing levels of LH and prolactin, inhibiting GnRH-induced LH release, and lowering testosterone production—effects that can be reversed by the melatonin receptor MT1 blocker, luzindole (Frungieri et al., 2017). Throughout testicular growth and development, melatonin shields the testes from local inflammation and ROS, affecting hormone production and testicular cell growth via its receptors. Secreted by the pineal gland and absorbed by the testes, melatonin regulates testicular function, including testosterone secretion, apoptosis, and autophagy (El-Shalofy et al., 2021).

*In vitro* treatment of bovine sertoli cells with melatonin has been shown to increase the expression of spermatogenesis-related genes, such as Cyclin D1, Cyclin E, Platelet-derived growth factor subunit A (PDGFA), desert hedgehog (Dhh), Occludin, and Claudin (Xu et al., 2020). In healthy animals, 6 months of melatonin administration has been linked to alterations in semen characteristics, possibly due to melatonin’s inhibition of aromatase. Furthermore, in a model of testicular ischemia-reperfusion, melatonin notably decreased the incidence of morphologically abnormal sperm (Heidarizadi et al., 2022). The effects of melatonin on reproductive system functions across various livestock species are further detailed in Table 4.

**TABLE 4:** Effects of melatonin in modulating reproductive system functions in animals.

Animal species	Effects of melatonin	Dose/Concentration	References
Pig	Modulates lipid metabolism in oocytes	10⁻⁹ M	Jin et al. (2017)
	Reduces ROS generation in oocytes and promotes mitochondrial function along with embryo development	500 nm/L	Niu et al. (2020)
	Enhances embryo quality	1 nM	Martinez et al. (2022)
	Increases acrosome integrity and semen viability	1 µM	Pezo et al. (2021)
	Regulates ATP metabolism and boosts antioxidant enzyme activity in sperm	1 µM	Lu et al. (2022)
Cattle/Buffalo	Promotes follicle enlargement and secondary oocyte growth	10⁻⁷ M	Lavrentiadou et al. (2023)
	Alters vaginal microbiome diversity (β diversity)	20 mg	Messman et al. (2021)
	Enhances the proliferation of theca cells while reducing steroid synthesis	1 µM	Feng et al. (2018)
	Facilitates oocyte maturation and growth	10⁻⁷ M	Tian et al. (2014)
	Protects granulosa cells by reducing oxidative stress and preventing cell death	100 M	Wang et al. (2023)
	Increases conception rates	0.24 mg/kg	Guo et al. (2021)
	Decreases ROS production in sperm, improves sperm viability, plasma membrane stability, mitochondrial, and acrosome integrity	10⁻³ M	Su et al. (2021)
	Influences Sertoli cell development and function	320 pg/mL	Yang et al. (2014)
Sheep/Goats	Reduces inflammation triggered by LPS in epididymal epithelial cells	10⁻⁷ M	Ge et al. (2019)
	Activates primordial follicles in ovaries	100 pg/mL	Barberino et al. (2022)
	Promotes the progression of transgenic embryos, enhancing transgenic success rates	10⁻⁷ M	Yao et al. (2022)
	Regulates ROS levels in testicular interstitial cells, boosts testosterone production	10 ng/mL	Ma et al. (2021)
	Enhances the activity of digestive enzymes like glucose amylase, isomaltase, and maltase	5 mg/day	Trotta et al. (2021)
	Improves sperm quality, maintains DNA integrity, and increases fertilization success	1 mM	Fang et al. (2020)

## 8 Productive performance

### 8.1 Growth and development

Melatonin significantly influences muscle development, fat deposition, and meat quality in livestock animals, with recent research emphasizing its diverse effects. Supplementing with melatonin improves growth performance and feed efficiency across various species. For example, it enhances body weight and average daily gain in broiler chickens (Fathi et al., 2023; Al-Jebory et al., 2024), and similarly boosts growth rates and muscle development in pigs (Xia et al., 2022; Chen et al., 2023) and cattle (Abulaiti et al., 2023).

Melatonin achieves these benefits by accelerating myoblast proliferation, enhancing the transcription of myogenic regulatory factors, and modulating levels of myogenin and embryonic myosin heavy chain (Han et al., 2017; Chen et al., 2019; Lu et al., 2020). These mechanisms improve muscle physiology, resulting in increased carcass weight and dressing percentage. Melatonin also affects fat deposition patterns, often leading to reduced fat accumulation and leaner meat, which is advantageous from a health perspective.

Melatonin impacts various meat quality attributes. It may improve tenderness by aiding muscle maturation and reducing stress-related muscle proteins. However, its effect on meat color is less consistent, with some studies noting minimal changes while others report potential alterations (Wang et al., 2007; Duan et al., 2019). Furthermore, melatonin can enhance water-holding capacity, contributing to juicier and higher-quality meat (Carcangiu et al., 2018).

Consumer acceptance of melatonin-treated meat largely depends on perceived health benefits, such as leaner meat and improved tenderness. Despite these benefits, some consumers may be cautious about hormonal treatments. Transparent labeling and clear communication regarding melatonin’s benefits and safety are crucial for building consumer trust (Owino et al., 2019).

The market value of melatonin-treated meat could increase if the treatment results in significant quality improvements, such as enhanced tenderness and leanness, justifying higher prices and appealing to premium market segments. Regulatory approvals and adherence to food safety standards are essential for the market viability of melatonin-treated meat. Overall, melatonin’s effects on growth, muscle development, and meat quality highlight its potential to enhance livestock productivity while meeting consumer and market demands.

### 8.2 Milk production

Melatonin has significant potential for enhancing milk production in livestock through various mechanistic pathways. In dairy cattle, melatonin administration affects milk yield by synchronizing circadian rhythms, optimizing the timing and efficiency of lactation processes (Garcia-Ispierto et al., 2013; Şahin et al., 2021). This hormone regulates the pineal gland to align the cows’ internal clocks with external environmental cues, which is crucial for maintaining consistent milk production.

Melatonin also mitigates the adverse effects of seasonal variations in daylight and temperature. By influencing cows’ photoperiodic responses, melatonin helps maintain stable milk yields throughout different seasons (Boztepe et al., 2022; Elhadi et al., 2022). Additionally, melatonin reduces stress levels in dairy cattle, a key factor since stress is known to inhibit milk production. The hormone’s stress-reducing properties enhance overall wellbeing and, consequently, improve lactation performance (Yao et al., 2020; Cosso et al., 2021).

Through its regulation of circadian rhythms, stabilization of seasonal variations, and stress reduction, melatonin contributes to more consistent and increased milk production, thereby enhancing dairy farm productivity. The intricate interplay between melatonin’s physiological effects and milk production highlights its potential as a valuable tool in optimizing livestock management practices.

## 9 Conclusion

Melatonin plays a critical and multifaceted role in enhancing livestock health, reproductive efficiency, and productive potential through both RM and receptor-independent actions, such as its potent antioxidant and free radical scavenging properties. As research continues to clarify its mechanisms, melatonin is poised to become an essential tool in promoting health across domestic animals. It’s ability to directly influence ovarian physiology, including steroid hormone synthesis, oocyte maturation, ovulation, and CL formation, while simultaneously mitigating oxidative stress, positions it as a valuable therapeutic agent for enhancing livestock fertility and advancing artificial reproductive technologies. Additionally, its capacity to alter systemic metabolites, such as amino acids, offers novel insights into enhancing livestock growth and productive performance. Although its application in animal science is still emerging, melatonin holds significant potential to transform livestock management by promoting resilience to environmental stressors and supporting sustainable animal husbandry practices.
